# Fabrication of selective and sensitive chemical sensor probe based on ternary nano-formulated CuO/MnO_2_/Gd_2_O_3_ spikes by hydrothermal approach

**DOI:** 10.1038/s41598-020-76662-6

**Published:** 2020-11-20

**Authors:** Mohammed M. Rahman, M. M. Alam, Abdullah M. Asiri, Firoz A. D. M. Opo

**Affiliations:** 1 Department of Chemistry, Faculty of Science, King Abdulaziz University, P.O. Box 80203, Jeddah, 21589 Saudi Arabia; 2 Center of Excellence for Advanced Materials Research (CEAMR), King Abdulaziz University, P.O. Box 80203, Jeddah, 21589 Saudi Arabia; 3 Department of Chemical Engineering and Polymer Science, Shahjalal University of Science and Technology, Sylhet, 3100 Bangladesh; 4 Department of Biomedical Science, College of Natural Sciences, Chosun University, Chosun, South Korea; 5 Phytochemistry Research Laboratory, Department of Pharmacy, University of Asia Pacific, Dhaka, Bangladesh

**Keywords:** Environmental monitoring, Two-dimensional materials

## Abstract

In this approach, thin spikes (NSs) of ternary nano-formulated mixed CuO/MnO_2_/Gd_2_O_3_ were synthesized by the hydrothermal approach for efficient detection of 3-methoxyphenyl hydrazine (3-MPHyd) chemical from various environmental samples. The NSs were systematically characterized by using XPS, EDS, TEM, FTIR, UV/vis, and XRD. The fabricated NSs onto the glassy carbon electrode (GCE) was successfully applied for the selective and sensitive detection of 3-MPHyd in the phosphate buffer system (PBS), which displayed the highest sensitivity, good selectivity with ultra-trace detection limit, high stability, good reproducibility, and quick response time. The real environmental samples were tested for validation from stand point of the ternary doped nanomaterials for sensing in the practical applications using by electrochemical method.

## Introduction

As strong reducing agents, generally the phenyl hydrazine and their derivatives are well-known, and they have various industrial applications such as blooming agent for plastics, corrosion inhibitor insecticides, and oxygen scavenger in the boiler as rocket fuel, photographic chemicals, aerospace fuel and explosives^1^. Due to exposure of these chemicals by human even at trace amount, may cause the hostile effect to human such as liver and kidney injury, haemolytic anaemia, skin irritation and dermatitis, etc. and the phenyl hydrazine and their derivatives are considered as a carcinogenic^2–4^. Then it is necessary to capture of 3-MPHyd at ultra-trace level to safe the human, environment, and the eco-system. The literatures have been described the detection methods of phenyl hydrazine and their derivatives such as fluorimetry, capillary electrophoresis, chromatography, spectrophotometry, photometry and photometric titrations, electrochemical technique^5–7^. But most of them, the electrochemical methods are widely implemented technique due to its high sensitivity, lower detection limit, greater simplicity, lower cost and long-term stability in chemical environment. Except few articles, the most of reported studies have been suffering in sensitivity, reproducibility and stability. To the phenyl hydrazine and their derivatives, the higher potential is required for GCE. Therefore, the modified GCE needs easy to fabricate for increasing the electrons transferring-rate as well as improving the sensitivity with lower detection limit^8^.

To reduce the over potential and increase the electron transfer rate, the various transition material such as copper oxides are used as detecting or capturing materials to the phenyl hydrazine and their derivatives^9^. Recently, the un-doped metal oxides (specially, transition) such as iron oxide, tungsten oxide, manganese oxide, and copper oxide are also studied as a detecting material of hydrazine and the derivatives of hydrazine^10^. Among them, MnO_2_ is the potential electron arbitrator as exhibited the high catalytic activity, precise surface area, lower resistances and pleasant electrochemical properties^11^. The CuO is a good electro mediator with better electro-chemical property with large surface area, which can enhance the electron transferring^12^. Generally, nanostructure materials have given significant attention for various organic as well as inorganic components, which is attracted owing to the binding affinity at optimum working conditions^13–16^.

A successful hydrazine derivative material with zinc oxide nano-urchins is reported and exhibited the good sensitivity as well as lower detection limit^17^. Another hydrazine derivate material based on silver-doped zinc oxide was reported, which was successful with high sensitivity and detection limit^18^. It is already established that CuO and MnO_2_ are efficient sensing elements to monitor the toxins for the safety of environment. Therefore, the ternary combination of CuO/MnO_2_/Gd_2_O_3_ NSs may be an effective detection material to detect environmental toxins due to the large active surface area in their interior or exterior surfaces. Various toxic analytes such as phenols and their derivatives, alcohol, acetone, ammonium hydroxide, dichloromethane, heavy-metal ions, hydrazine, aliphatic and aromatic compounds have been detected by various compositions of metal oxide/sulphides with the doped or un-doped, mixed/dual combinations^19^. The nanostructure materials prepared by sol–gel method have been shown elsewhere with the higher surface area for good adsorption of chemicals and ions^20–23^. From the specific advantage of the active surface area with ternary metal oxides compared to single or dual/doped combination, it is highly demanded to introduce new doped materials with metal oxides.

Generally, mixed doped metal oxide exhibits promising results for the detection and quantification of hazardous chemicals by various detection approaches. Besides this, as enhancing of electrochemical properties of CuO/MnO_2_/Gd_2_O_3_ NS nanostructure material by converting it to a doped mixed oxides with other materials is also cost-effective. Here, CuO/MnO_2_/Gd_2_O_3_ NS nanostructured materials have employed a great deal of consideration due to their chemical, structural, physical, and optical properties in terms of large-active surface area, high-stability, and high porosity. These properties are directly depend on the structural morphology prepared by reactant precursors for making the CuO/MnO_2_/Gd_2_O_3_ porous materials in the basic medium at low-temperature. This CuO/MnO_2_/Gd_2_O_3_ NSs material was synthesized by a facile solution method using NaOH solution. This technique has several advantages including facile preparation, accurate control of reactant temperature, easy to handle, one-step reaction. Optical, morphological, electrical, and chemical properties of the CuO/MnO_2_/Gd_2_O_3_ NS nanomaterials are of huge significance from the scientific aspect, compared to other undoped materials. The non-stoichiometry, mostly oxygen vacancies, Gd_2_O_3_ makes it conducting nature in the doped nanostructured materials^24–29^. The formation energy of oxygen vacancies and metal interstitials in the semiconductor is very low and thus these defects form eagerly, resulting the increased conductivity of CuO/MnO_2_/Gd_2_O_3_ NS materials compared to other undoped materials. CuO/MnO_2_/Gd_2_O_3_ NS materials have also attracted considerable interest due to their potential applications in fabricating optoelectronic, electro-analytical, selective detection of assays, chemical sensor devices, hybrid-composites, electron-field emission sources for emission exhibits, biochemical detections, and surface-enhanced Raman properties, etc. CuO/MnO_2_/Gd_2_O_3_ NS material offers improved performance due to the large-active surface area, which increased conductivity and current responses of the CuO/MnO_2_/Gd_2_O_3_ NSs/Nafion/GCE assembly during the electrochemical investigation.

The most reliable hydrothermal process was applied to synthesize CuO/MnO_2_/Gd_2_O_3_ NSs. The fabricated NSs were practice for the coating onto a GCE, and the binding properties between these were enhanced by the addition of nafion solution. The resulted GCE was investigated for efficient capturing of 3-MPHyd in the aqueous media. The analytical capturing performances were studies very carefully, and the outcome of the 3-MPHyd chemical material was high sensitivity with the low detection limits. Here, it is introduced a significant material in this approach for selective and sensitive monitoring and capturing of selective 3-MPHyd with CuO/MnO_2_/Gd_2_O_3_ NSs sensor probe.

## Experimental section

### Materials and method

In this research work, the required chemicals were used as received without further purification. The inorganic salts of transition metals such as copper(II) chloride (CuCl_2_), manganese(II) chloride (MnCl_2_), gadolinium(III) chloride, and ammonium hydroxide (NH_4_OH) were purchased from well-known Sigma-Aldrich, which was deployed to prepare ternary CuO/MnO_2_/Gd_2_O_3_ NSs. As a part of these studies, the analytical grade chemicals 2,4-DNP (2,4-dinitrophenol), 3,4-DAT (3,4-diaminotoluene), pyridine, BH (benzaldehyde), 3-chlorophenol, THF (tetrahydrofuran), methanol, 3-MPHyd (3-methoxyphenylhydrazine), AH (ammonium-hydroxide), coating agent nafion (in 5% ethanol), NaH_2_PO_4_, and Na_2_HPO_4_ were also bought from the Sigma-Aldrich and deployed as-received. For the details characterization of synthesized CuO/MnO_2_/Gd_2_O_3_ NSs, conventional XRD, XPS, FTIR, UV/Vis and FESEM were implemented to evaluate the structural, crystalline, functional, optical, morphological and elemental analyses. The reliable current versus potential (electrochemical method) was used to determine 3-MPHyd with active CuO/MnO_2_/Gd_2_O_3_ NSs material by using Keithley electrometer (6517A, USA) at room conditions.

### ***Hydrothermally synthesis of CuO/MnO***_***2***_***/Gd***_***2***_***O***_***3***_*** NSs***

The inorganic salts copper chloride (CuCl_2_), manganese chloride (MnCl_2_), gadolinium chloride (GdCl_3_) and alkali ammonium hydroxide (NH_4_OH) were used to prepare CuO/MnO_2_/Gd_2_O_3_ NSs by solvo/hydrothermal method at low temperature. The solvothermal process was widely used efficient method to fabricate nanomaterials of metal oxides, and the resultant guest or doped metal oxides are smaller in size as well as phase formation. Following this method, 100.0 mL of 0.10 M CuCl_2,_ 100.0 mL of 0.10 M MnCl_2_, 100.0 mL of 0.10 M GdCl_3_ and 100.0 mL of 0.10 M NH_4_OH were prepared in a different four 200.0 mL beaker with de-ionized water and resultant solutions were kept with continuous magnetic stirring. Another 250.0 mL of conical flask was taken and 50.0 mL of each prepared metallic salt solution was added. Then the mixture was shacked with continuous magnetic stirring onto the hot plate. To obtain the co-precipitation of metal hydroxides, the prepared 0.10 M NH_4_OH was added slowly and at the pH value 10.5, all metal hydroxides were precipitated out in conical flask. Then the total solution was kept at 80 °C on the hot plate with continuous magnetic stirring around 6 h. As-prepared participate of metal hydroxides were washed thoroughly by de-ionized water and kept it to dry at room condition for overnight. Consequently, the powdered sample was heated for calcination at 510 °C for 6 h. Under higher temperature, the metal oxides is transform to crystalline metal oxide i.e., CuO/MnO_2_/Gd_2_O_3_ nanostructure shapes, which contains the higher metallic-ions. The prepared material was properly grained into fine powder of nano-sized materials for details characterization. The following reactions may happen:

In the aqueous medium:

1 $${\text{NH}}_{4} {\text{OH}}_{{({\text{s}})}} \to {\text{ NH}}_{{4({\text{aq}})}}^{ + } + {\text{ OH}}_{{({\text{aq}})}}^{ - }$$

2 $${\text{CuCl}}_{{2\left( {\text{s}} \right) }} \to {\text{Cu}}_{{\text{(aq)}}}^{2 + } + 2{\text{Cl}}_{{\text{(aq)}}}^{ - }$$

3 $${\text{MnCl}}_{{2 \, ({\text{s}})}} \to {\text{ Mn}}_{{({\text{aq}})}}^{2 + } + \, 2{\text{Cl}}_{{({\text{aq}})}}^{ - }$$

4 $${\text{GdCl}}_{{3({\text{s}})}} \to {\text{ Gd}}_{{\text{(aq)}}}^{3 + } + \, 3{\text{Cl}}_{{({\text{aq}})}}^{ - }$$

5 $$\begin{aligned} & {\text{NH}}_{{4({\text{aq}})}}^{ + } + 7{\text{OH}}_{{({\text{aq}})}}^{ - } + {\text{Cu}}_{{({\text{aq}})}}^{2 + } + {\text{Mn}}_{{({\text{aq}})}}^{2 + } + {\text{ Gd}}_{{({\text{aq}})}}^{3 + } + {\text{ Cl}}_{{({\text{aq}})}}^{ - } \\ \quad &\quad \to {\text{Cu}}\left( {{\text{OH}}} \right)_{2} {\text{/Mn}}\left( {{\text{OH}}} \right)_{2} {\text{/Gd}}\left( {{\text{OH}}} \right)_{{3({\text{aq}})}} \downarrow \, + {\text{ NH}}_{{4}} {\text{Cl}}_{{\text{(aq)}}}. \\ \end{aligned}$$

In furnace:

6 $${\text{Cu}}\left( {{\text{OH}}} \right)_{2} {\text{/Mn}}\left( {{\text{OH}}} \right)_{2} {/}2{\text{Gd}}\left( {{\text{OH}}} \right)_{{3({\text{aq}})}} + {\raise0.7ex\hbox{$1$} \!\mathord{\left/ {\vphantom {1 2}}\right.\kern-\nulldelimiterspace} \!\lower0.7ex\hbox{$2$}}\,{\text{O}}_{2} \to {\text{ CuO/MnO}}_{2} {\text{/Gd}}_{{2}} {\text{O}}_{{3({\text{s}})}} + \, 5{\text{H}}_{2} {\text{O}} \uparrow .$$

The *Ks* was low (*Ks* = 2.2 × 10^–20^ in Cu(OH)_2_, 2.0 × 10^–13^ for Mn(OH)_2_ and 1.88 × 10^–23^ for Gd(OH)_3_^30^. Metal ions were precipitated out quantitatively as various oxides. The crystal formation was happened initially, where an aggregation to the Gd(OH)_3_ was started. In the reaction system, pH was continued to enhance, then the Cu(OH)_2_ was started to precipitate, which was re-aggregated onto the Gd(OH)_3_ crystallites. Further increasing of pH, Mn(OH)_2_ is also participated out to form aggregation with other two metal hydroxides. The formation of NSs is similar with the previously reported article^31^. The synthesized NSs were characterized in terms of elemental composition, crystallinity, optical property, morphology, structure, and functional properties. Later, CuO/MnO_2_/Gd_2_O_3_ NSs were applied to detect 3-MPHyd by reliable electrochemical method at room conditions. This is the first time, the produced CuO/MnO_2_/Gd_2_O_3_ NSs were implemented for the selective determination of 3-MPHyd for environmental safety by electrochemical method.

### ***Fabrication of CuO/MnO***_***2***_***/Gd***_***2***_***O***_***3***_***/Nafion/GCE sensor probe***

**Figure 1:**
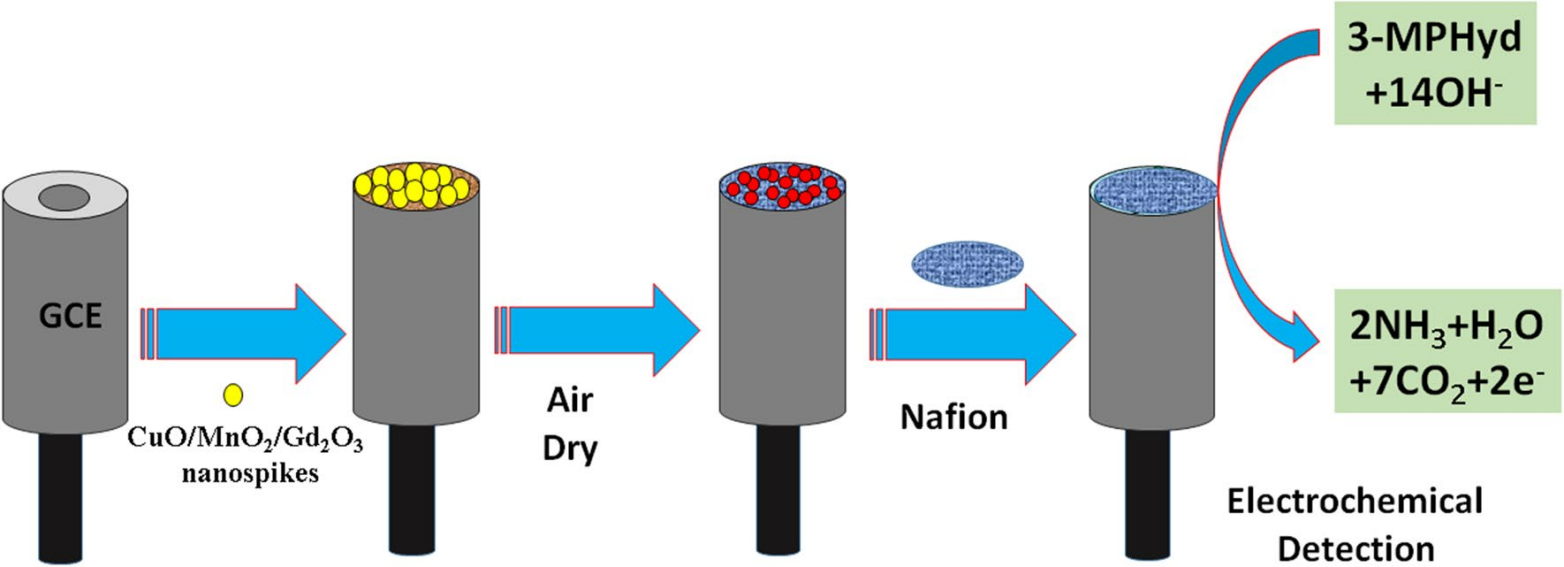
Fabrication of GCE with ternary doped CuO/MnO_2_/Gd_2_O_3_ NSs by using 5% nafion conducting coating binder.

The ternary doped materials based on the NSs of CuO/MnO_2_/Gd_2_O_3_ was successively implemented to determine the target environmentally unsafe 3-MPHyd in reaction medium. To prepare the working electrode for 3-MPHyd detection, the ethanolic slurry of CuO/MnO_2_/Gd_2_O_3_ NSs was put onto the GCE. Platinum wire (Pt-wire) was used as a counter electrode. The dispersed materials was attached between NSs of ternary metal oxides and GCE by air dry initially. It was fabricated on the flat GCE and dried in air for complete thin-film formation. Later, after drying completely, 1.0 μL of 5.0% Nafion (ethanolic) was dropped onto the fabricated electrode surface and waited until dry it completely. Here, nafion is used as a chemical glue for the stable attachment of ternary materials onto the surface of flat-GCE. Then the dried fabricated electrode was used as working electrode in this investigation. The electrochemical cell was composed by CuO/MnO_2_/Gd_2_O_3_/binders/GCE sensor probe as working electrode. The fabrication scheme is presented in the Fig. 1. The target analyte 3-MPHyd was used to prepare the solution in di-ionized water on the concentration range from 1.0 mM to 1.0 pM and this formulated solutions of 3-MPHyd (lower to higher concentration) were investigated into electrochemical cell (chemical material). Then the linearity was calculated from the linear plot by using regression co-efficient (r^2^). The other analytical properties of 3-MPHyd chemical sensor such as LDR and LOD were estimated according to ratio of 3 N/S.

## Results and discussion

### Materials properties

**Figure 2:**
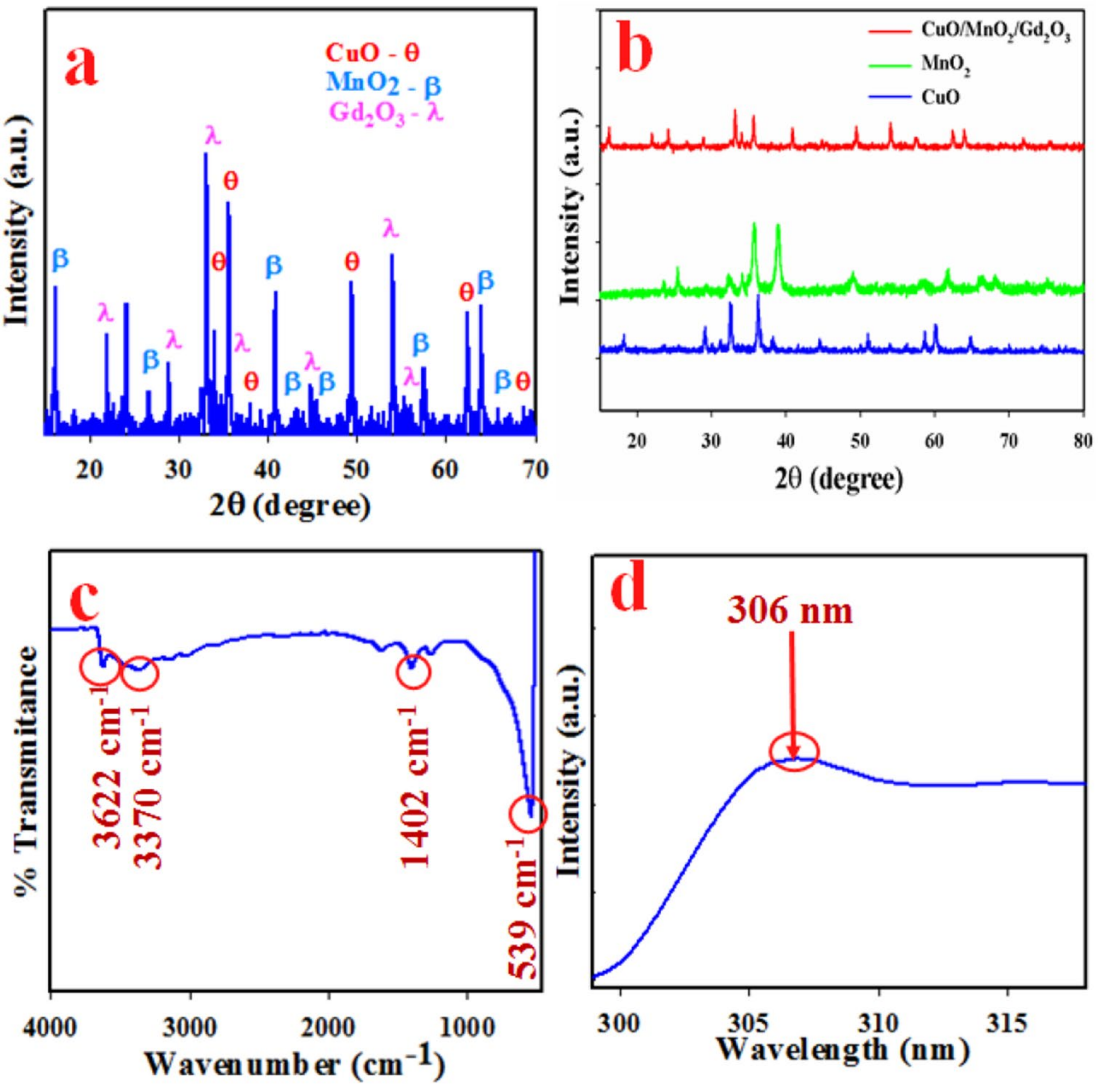
Structural and optical characterization of ternary doped CuO/MnO_2_/Gd_2_O_3_ NS materials. (**a**) XRD pattern of CuO/MnO_2_/Gd_2_O_3_ NSs, (**b**) XRD of CuO, MnO_2_, Gd_2_O_3_ NSs, (**c**) FTIR of CuO/MnO_2_/Gd_2_O_3_ NSs, and (**d**) UV absorbance of the CuO/MnO_2_/Gd_2_O_3_ NSs.

The crystallographic studies of prepared CuO/MnO_2_/Gd_2_O_3_ NSs were carried out by the implementation of XRD. As shown in Fig. 2a, the fabricated NSs were consisting of diversified crystalline mediums of CuO, MnO_2_ and Gd_2_O_3_. The resultant XRD spectra is represented the Bragg planes of CuO indices as θ such as (110), (111), (200), (202), (113), and (022), which has the similarities with JCPDS No. 72-0629 and earlier published articles^32,33^. The other diffracted peaks created from MnO_2_ indices as ß are (101), (110), (111), (200), (210), (211), (002) and (310). This XRD pattern of MnO_2_ is agreed with the JCPDS Card, No. 24-0735 and literatures^34,35^. The additional sharpest peaks of Gd_2_O_3_ indices as λ are (211), (222), (411), (442), (611) and (622), which are agreed with those reported for gadolinium oxide nanoparticles^36^ and JCPDS No. 43-1015. There is a provision for measuring the crystallinity by the XRD pattern through Scherer’s expectation (vii).

7 $${\text{D}} = \, 0.9\uplambda{\text{/}}(\upbeta \cos {\uptheta})$$

where λ is wavelength (1.5418 Å) and β is width at half, according to the apex peak, and θ is the diffracting edge^37^. Here, the determined crystallinity was 43.31 nm. Additionally, it is also compared the XRD spectrum of individual CuO, MnO_2_, and CuO/MnO_2_/Gd_2_O_3_ NSs and presented in the Fig. 2b. FTIR investigation of synthesized CuO/MnO_2_/Gd_2_O_3_ NSs is depicted in Fig. 2c and obtained peaks are at 539, 1402, 3370 and 3622 cm^−1^. The main characteristic absorption peak is at 539 cm^−1^ which is corresponding to Mn–O or Cu–O stretching modes^38^ and the identical peak at 1402 cm^−1^ is responsible for C−O stretching^39^. The other two peaks at 3370 and 3622 are corresponding to the OH group^40^. The visual spectra of CuO/MnO_2_/Gd_2_O_3_ NSs were completed at the range between 200.0 and 800.0 nm wavelengths. As judged from Fig. 2d, the maximum intensity was inspected at 306 nm and this was the evaluated absorption band of prepared CuO/MnO_2_/Gd_2_O_3_ NSs^41^. According to the Eq. (8), the defined energy band-gap (E_bg_) was 4.05 eV of the CuO/MnO_2_/Gd_2_O_3_ NSs.

$$E_{bg} ({\text{eV}}) = \frac{1240}{\lambda }$$

where E_bg_ = energy band-gap and λ = maxima absorbed area.

### Structural analyses

**Figure 3:**
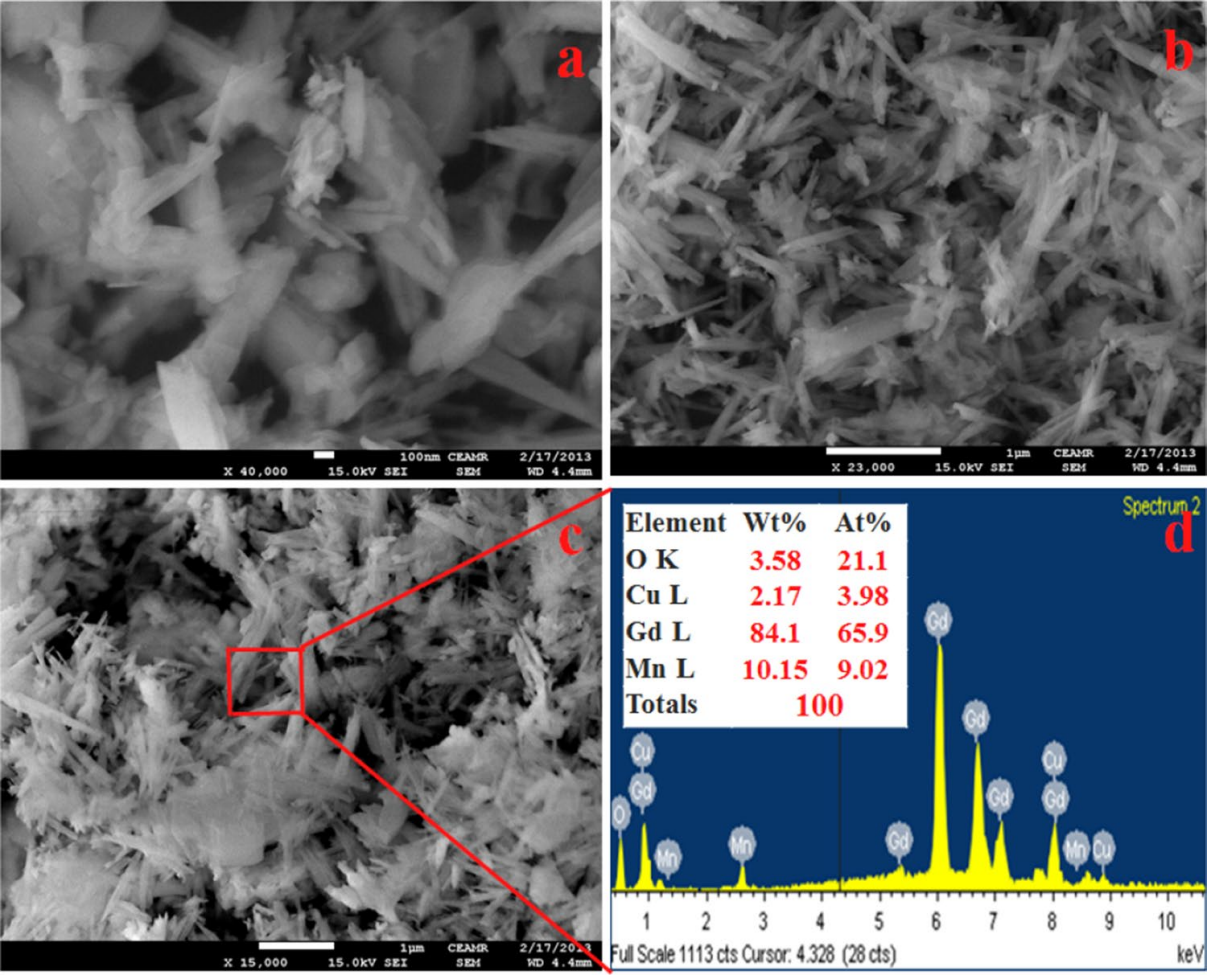
FESEM analysis with different morphologies (**a**,**b**) and elemental quantification of hydrothermally prepared CuO/MnO_2_/Gd_2_O_3_ NSs (**c**,**d**).

The structural analyses of the fabricated NSs were evaluated by FESEM images. The resultant FESEM of CuO/MnO_2_/Gd_2_O_3_ NSs from higher to lower magnifying images are depicted in Fig. 3a,b and it is clearly shown a uniform aligned of CuO/MnO_2_/Gd_2_O_3_ nanospikes. From Fig. 3c,d, the EDS define of CuO/MnO_2_/Gd_2_O_3_ demonstrated the existence of Cu, Mn, Gd and O and the elemental arrangements of calcined co-doped metal oxides are nanospikes in shape. The atomic compositions (wt%) of CuO/MnO_2_/Gd_2_O_3_ NSs are as O 3.58%, Cu 2.17%, Gd 84.1% and Mn 10.15%. Any additional peaks are not detected, which is associated with impurities. Therefore, the synthesized NSs are consisted only Cu, Mn, Gd and O.

### Binding energy analysis

**Figure 4:**
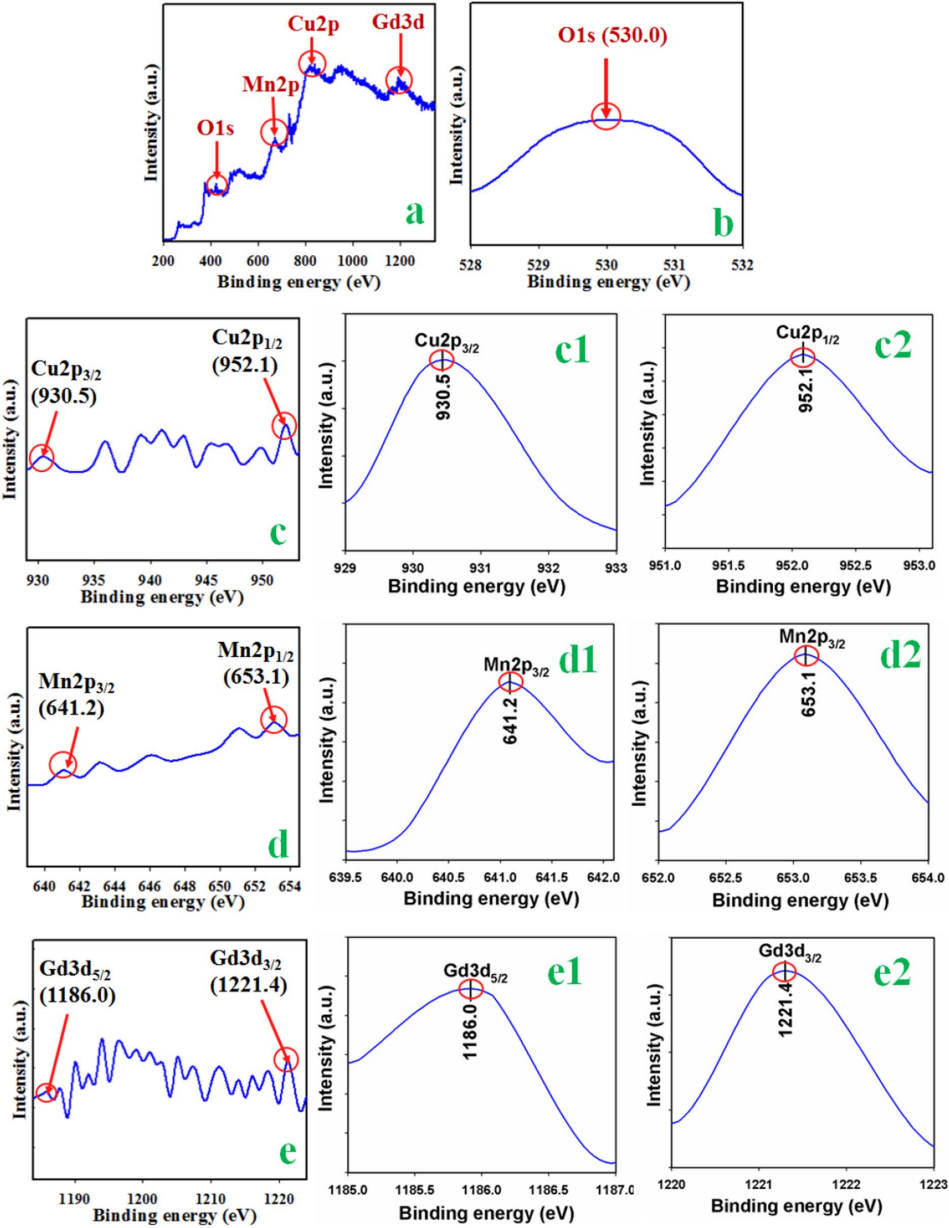
XPS study of doped CuO/MnO_2_/Gd_2_O_3_ NSs. (**a**) XPS spectrum, (**b**) O1*s* level, (**c**) spin–orbit Cu2*p* level, (**c1**,**c2**) magnified peak of Cu2*p* level, (**d**) spin–orbit of Mn2*p* level, (**d1**,**d2**) magnified peak of Mn2*p* level, (**e**) spin–orbit of Gd3*d* level, and (**e1**,**e2**) magnified peaks of Gd3*d* level.

The XPS is defined in Fig. 4 are also investigated to evaluate the chemical composition, electronic and valance states of prepared CuO/MnO_2_/Gd_2_O_3_ NSs. As it is shown the full spectrum (Fig. 4a), Cu2*p*, Mn2*p* Gd3*d* and O1*s* core level of spin orbitals are investigated. The observed O1*s* peak at 530.0 eV, which was presented in Fig. 4b and assigned to the O^2−^^42,43^. The Cu2*p* spin orbital ruptures into Cu2*p*_3/2_ and Cu2*p*_1/2_ as depicted in Fig. 4c. Here, it displays the high resolution spectrum of Cu 2*p*, separated into Cu 2*p*_3/2_ and Cu 2*p*_1/2_ at 930.5 eV and 952.1 eV, respectively (Fig. c1 and c2). The distance between these Cu 2*p* main peaks positions is 21.6 eV, which agrees well with previous reports about CuO spectrum. It is also denoted to the existence of Cu^2+^ chemical state as an indication of the formation of CuO, which is matched to the reported literatures^44–52^. Moreover, additional confirmation of CuO state was seen with the broad satellite peaks at a higher binding energy than the main peaks. The main peak of Cu 2*p*_3/2_ at 930.5 eV was accompanied by satellite peaks on the higher binding energy side at 939.2 eV, 941.5 eV and 943.1 eV, which suggests the existence of CuO. From the full spectrum, we can clearly see that the main peak of Cu 2*p*_1/2_ at 952.1 eV, which also confirms the presence of CuO. The XPS spectrum is also exhibited the two major peaks of Mn2*p* orbital and the resultant spectrum of Mn2*p* is represented in Fig. 4d. As evaluated, the spin energy of Mn2*p*_3/2_ (641.2 eV) and Mn2*p*_1/2_ (653.1 eV) are also the adjacent position with the reported data for MnO_2_^45,46^, which is presented separately in Fig. 4d1,d2. In Gd3*d* spectra (Fig. 4e), two peaks are found, where the binding-energy of strong peak at ~ 1186.0 eV is responsible for Gd3*d*_5/2_, and the binding-energy of weak peak at ~ 1221.4 eV is responsible for Gd3*d*_3/2_. It is associated with the oxidation state of Gd^3+^, which is represented in the Fig. 4e1, e2^49,50^.

### ***Sensing of 3-MPHyrd by CuO/MnO***_***2***_***/Gd***_***2***_***O***_***3***_*** NSs***

The proposed application of 3-MPHyrd chemical material was to detect 3-MPHyrd in environmental real samples. The chemical material based on CuO/MnO_2_/Gd_2_O_3_ NSs is first stage, and any report regarding phenyl hydrazine analyte is not available. Here the conducting binder of nafion is used to enhance the stability with high conductivity and electron transfer^51,52^. During the sensing performances of proposed 3-MPHyd chemical material, electrochemical responses of CuO/MnO_2_/Gd_2_O_3_ NSs were increased with increasing of 3-MPHyd amount. In presence of higher concentration of target analyte, the resultant current is gradually increased by oxidation of 3-MPHyd onto CuO/MnO_2_/Gd_2_O_3_ NSs. In this investigation, the flat GCE was coated with the ethanolic slurry of CuO/MnO_2_/Gd_2_O_3_ NSs and dried it in the ambient temperature. Later, the fabricated CuO/MnO_2_/Gd_2_O_3_ NSs/Nafion/GCE electrode was employed to sensing the 3-MPHyd by electrochemical approach at room conditions. The 3-MPHyd sensing mechanism onto CuO/MnO_2_/Gd_2_O_3_ NS probe is based on the ternary metal oxides, owing to adsorption/absorption of aqueous oxygen onto the exterior or interior surface of CuO/MnO_2_/Gd_2_O_3_ NSs, according to the dissolved O_2_ in bulk-solution or surface-air of the surrounding atmosphere (Eqs. 9–11). These reactions are taken place in bulk-solution or air/liquid interface or surrounding air due to the low carrier concentration, which probably increased the resistance of ternary material surface as well as decreased the conductivity^53–56^. The anlayte 3-MPHyd sensitivity towards CuO/MnO_2_/Gd_2_O_3_ NSs could be attributed to the high oxygen deficiency and defect the density leads to increase the oxygen adsorption for form active as O_2_^-^, then O^-^ and finally OH^-^. Larger the amount of oxygen adsorbed on the mixed CuO/MnO_2_/Gd_2_O_3_ NSs surface, larger would be the oxidizing capability and faster would be the oxidation of 3-MPHyd.

9 $${\text{e}}^{ - } ({\text{onto}}\,{\text{CuO/MnO}}_{2} {\text{/Gd}}_{{2}} {\text{O}}_{3} \,{\text{NSs}}\,{\text{surface }}) + {\text{ O}}_{2} \to {\text{ O}}_{2}^{ - }$$

10 $${\text{e}}^{ - } \,({\text{onto}}\,{\text{CuO/MnO}}_{2} {\text{/Gd}}_{2} {\text{O}}_{3} \,{\text{NSs}}\,{\text{surface}}) \, + {\text{ O}}_{2}^{ - } \to \, 2{\text{O}}^{ - }$$

11 $${\text{O}}^{ - } \,({\text{onto}}\,{\text{CuO/MnO}}_{2} {\text{/Gd}}_{2} {\text{O}}_{3} \,{\text{NSs}}\,{\text{surface}}) + {\text{ H}}_{2} {\text{O }} \to \, 2{\text{OH}}^{ - } .$$

**Figure 5:**
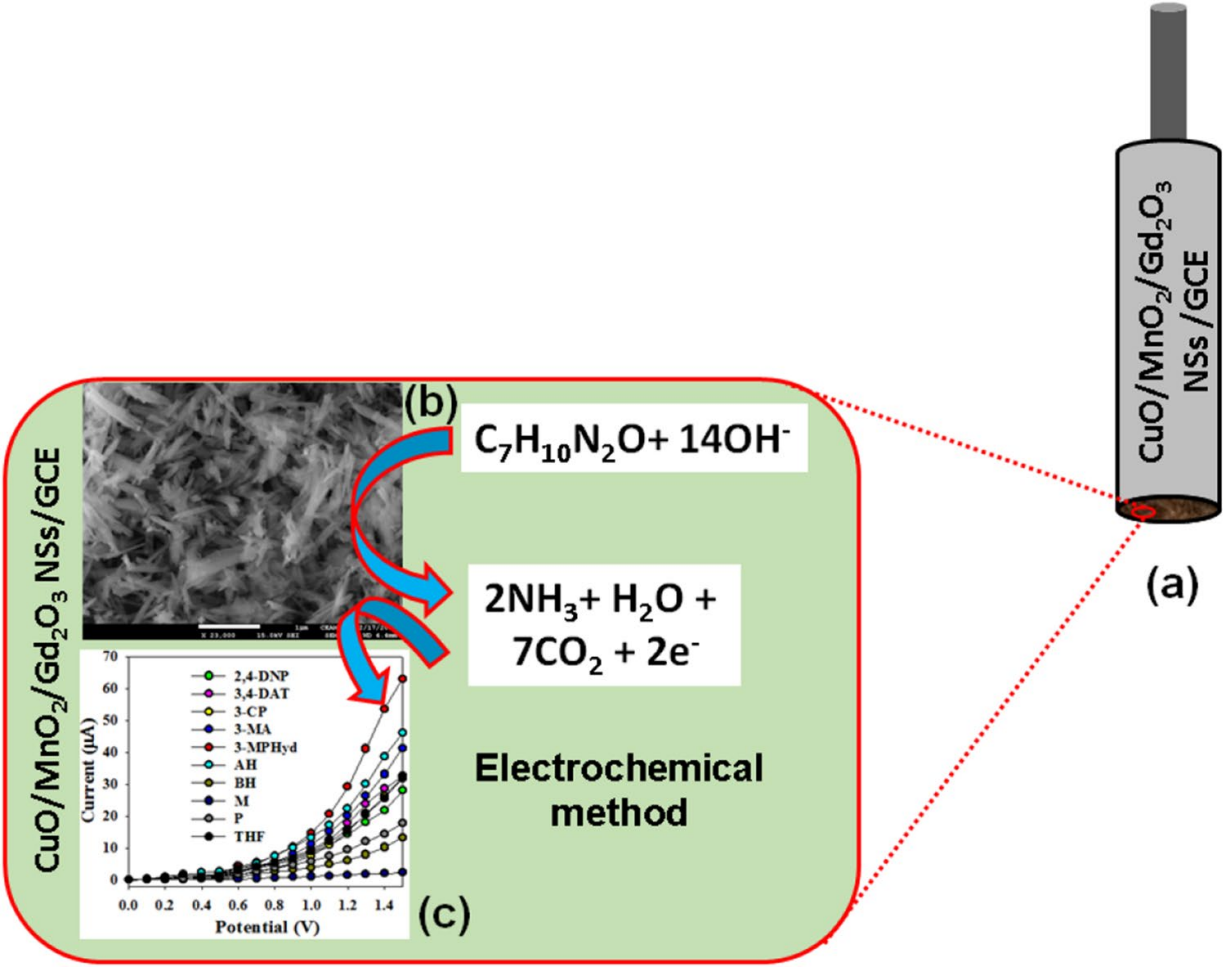
Schematic diagram of sensor fabrication and probable mechanism. Possible bonding mechanism of 3-MPHyd by capturing of the thin nanospikes in electrochemical approach. (**a**) GCE fabrication with CuO/MnO_2_/Gd_2_O_3_ NSs and conducting nafion-coating binders; (**b**) Possible mechanism of 3-MPHyd capturing onto CuO/MnO_2_/Gd_2_O_3_ NSs surfaces; (**c**) 3-MPHyd detection by electrochemical method using CuO/MnO_2_/Gd_2_O_3_ NSs modified GCE.

The pictographic representation (Fig. 5a) and mechanism (Fig. 5b) of the CuO/MnO_2_/Gd_2_O_3_ NSs modified electrode of 3-MPhyd chemical material is depicted in Fig. 5. As clarified in Fig. 5c, the electrochemical signalling data was illustrated with prepared NS of CuO/MnO_2_/Gd_2_O_3_, which is exhibited the higher current response compared to pure CuO and MnO_2_. Here, the oxidation reaction of 3-MPHyd onto surface of CuO/MnO_2_/Gd_2_O_3_ NSs/Nafion/GCE into the buffer system is proposed and presented below according to Eq. (12). According to the electrochemical oxidation process, targeted 3-MPHyd molecule is oxidized, hence released the electrons. Thus it is increased the electrochemical response with the CuO/MnO_2_/Gd_2_O_3_ NSs/Nafion/GCE sensor probe in the electrochemical system during measurement of resultant current. As a result, in contact with the CuO/MnO_2_/Gd_2_O_3_ NS surface, the target analyte 3-MPHyd is directly oxidized by releasing two electrons onto the sensor surface of CuO/MnO_2_/Gd_2_O_3_ NSs/Nafion/GCE probe, which is measured during the electrochemical measurement at room conditions. During the oxidation of 3-MPHyd, the resultant current is significantly increased by producing ammonia, water, and carbon dioxide into the electrochemical process.

12 $${\text{C}}_{7} {\text{H}}_{10} {\text{N}}_{2} {\text{O}}\, \, (3{\text{-MPHyd}}) \, + \, 14{\text{OH}}^{ - } \,({\text{onto}}\,{\text{CuO/MnO}}_{2} {\text{/Gd}}_{2} {\text{O}}_{3} \,{\text{NS}}\,{\text{surface}}) \to {\text{ NH}}_{{3}} + {\text{ H}}_{2} {\text{O}} + \, 7{\text{CO}}_{2} + \, 2{\text{e}}^{ - }$$

**Figure 6:**
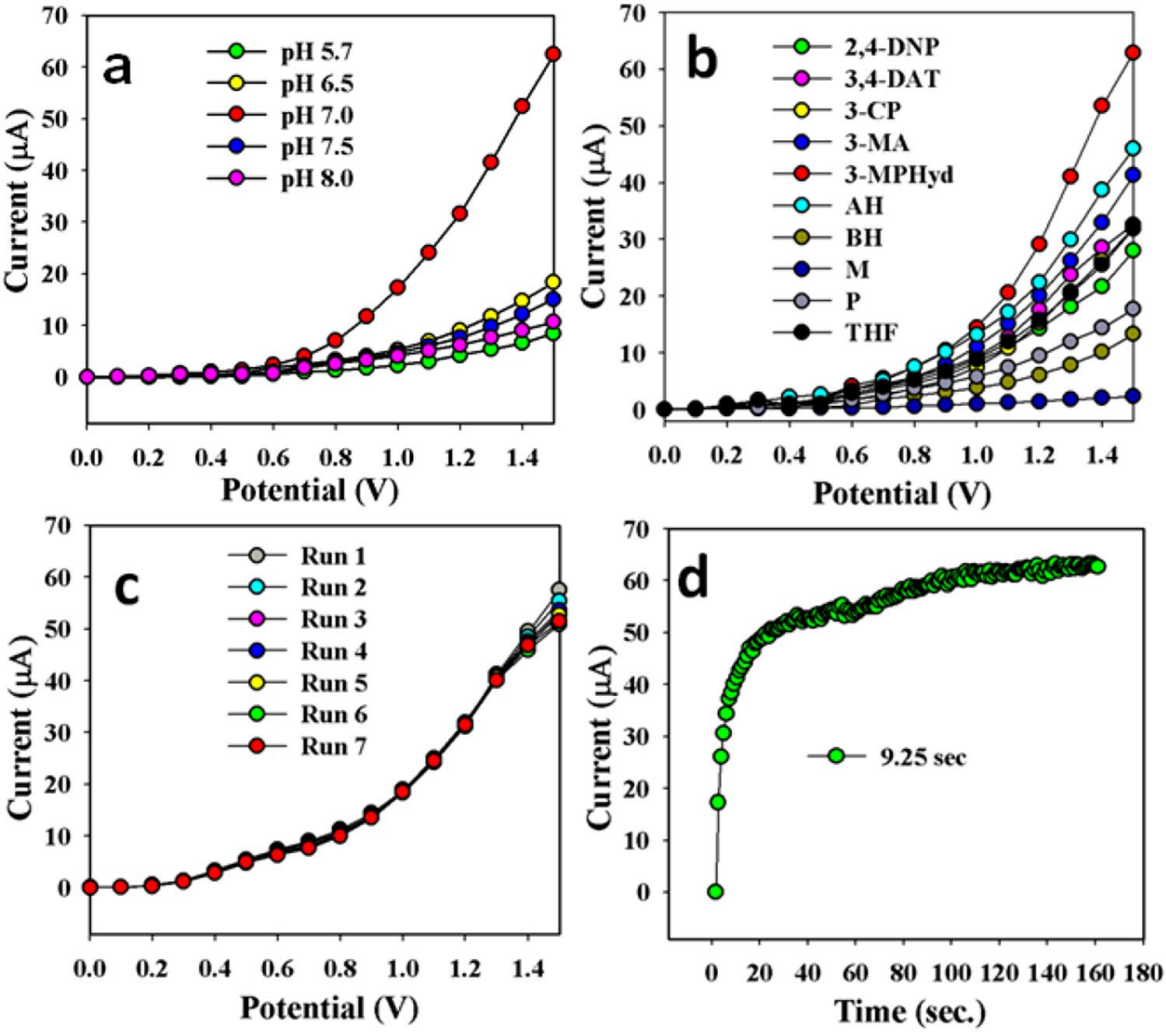
Optimization of 3-MPHyd chemical material based on CuO/MnO_2_/Gd_2_O_3_ NSs/binder/GCE. (**a**) pH conditioning, (**b**) selectivity, (**c**) repeatability, and (**d**) response time. Analyte concentration: 0.10 nM; Holding time: 1.0 s; Electrochemical method; Potential ranges: 0 to + 1.5 V.

The synthesized CuO/MnO_2_/Gd_2_O_3_ NSs/binder/GCE is not equally given electrochemical response in the full range in buffer system. The invented working electrode was investigated in alkaline and acidic media, and it was observed that the chemical material was exhibited the maximum electrochemical response in pH 7.0. The pH optimization performance is illustrated in Fig. 6a. To obtain the selectivity, the fabricated working electrode based on CuO/MnO_2_/Gd_2_O_3_/binder/GCE was performed in presence of various toxins such as 2,4-DNP (2,4-dinitrophenol), 3,4-DAT, pyridine, BH, 3-CP, THF, methanol, 3-MPHyd, and AH. As it is depicted in Fig. 6b, 3-MPHyd was displayed with the highest electrochemical responses. Also the most important analytical characteristic of chemical material is the ability to reproducible performance repeatedly. This performance of sensor was executed in 0.1 nM concentration of 3-MPHyd solution. The outstanding reproducibility was observed, which is shown in Fig. 6c.

**Figure 7:**
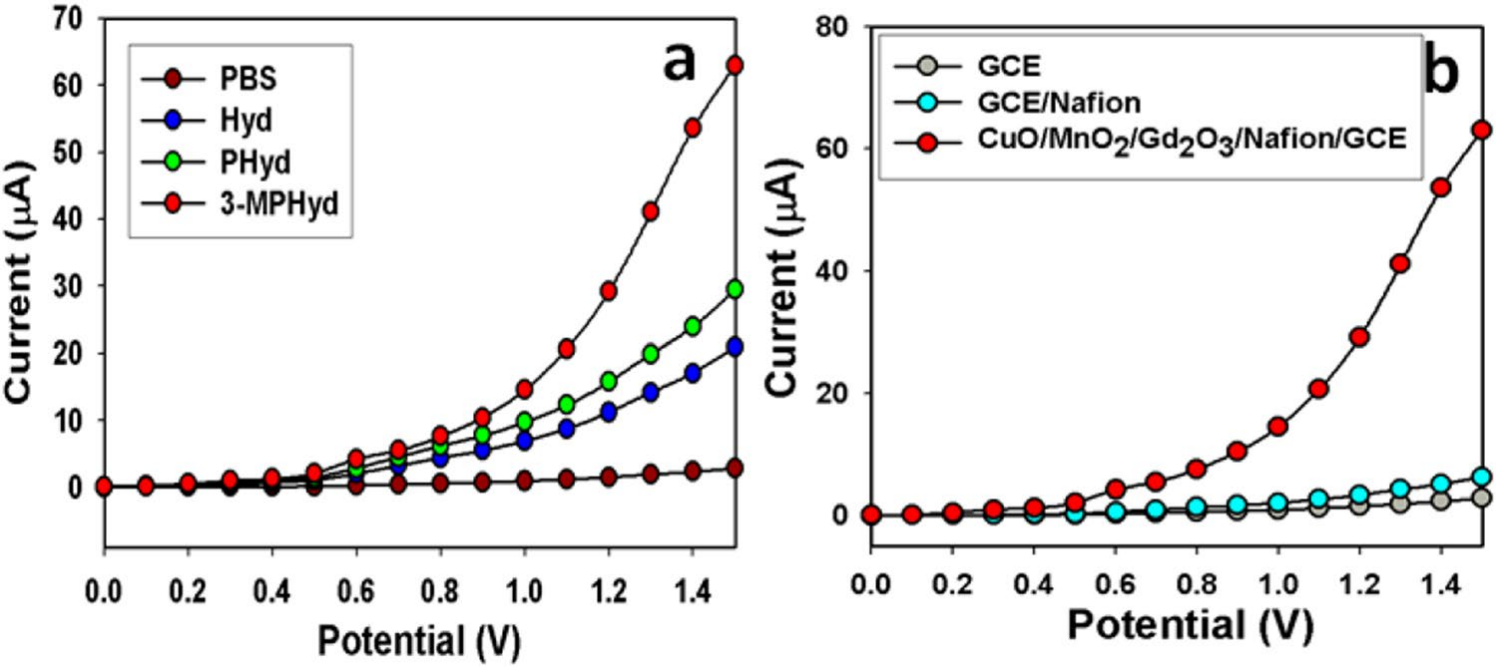
Control experiment of fabricated chemical sensor probe. (**a**) Comparative study of different derivatives of hydrazine such as hydrazine, phenylhydrazine, 3-methoxyphenlyhydrazines. (**b**) Comparative study of various fabricated GCE, GCE/Naifon, CuO/MnO_2_/Gd_2_O_3_/Nafion/GCE electrodes. Analyte concentration: 0.1 nM; Holding time: 1.0 s; Electrochemical method; Potential ranges: 0 to + 1.5 V.

The calculated RSD (relative standard deviation) is 1.10%, which is measured at + 1.0 V. The response time with the fabricated working electrode based on CuO/MnO_2_/Gd_2_O_3_/binder/GCE is 9.25 s. It was evaluated under the certain amount of 0.1 nM of 3-MPHyd solution, which is shown in Fig. 6d. The fabricated sensor is very fast response towards the target analyte with CuO/MnO_2_/Gd_2_O_3_/binder/GCE sensor probe by electrochemical method. After the 9.25 s, signal become sensor response become stable and flat, due to the saturation of contact surface with target analyte. The fabricated materials CuO/MnO_2_/Gd_2_O_3_/binder/GCE was studied and compared in presence of various hydrazine derivatives (Fig. 7a). It was found that 3-MPHyd shows the highest electrochemical response (Fig. 7a) compared to blank solution (without 3-MPHyd), only hydrazine and phenylhydrazine derivatives in the identical conditions. Additionally, a control experiment has been performed with the only GCE, GCE/Nafion, and GCE/Nafion/CuO/MnO_2_/Gd_2_O_3_ electrodes in the identical conditions in presence of target 3-MPHyd chemical, which is presented in the Fig. 7b. It is observed that the CuO/MnO_2_/Gd_2_O_3_ fabricated glassy carbon electrode is showed the highest electrochemical current compared to only GCE and GCE/Nafion electrodes.

**Figure 8:**
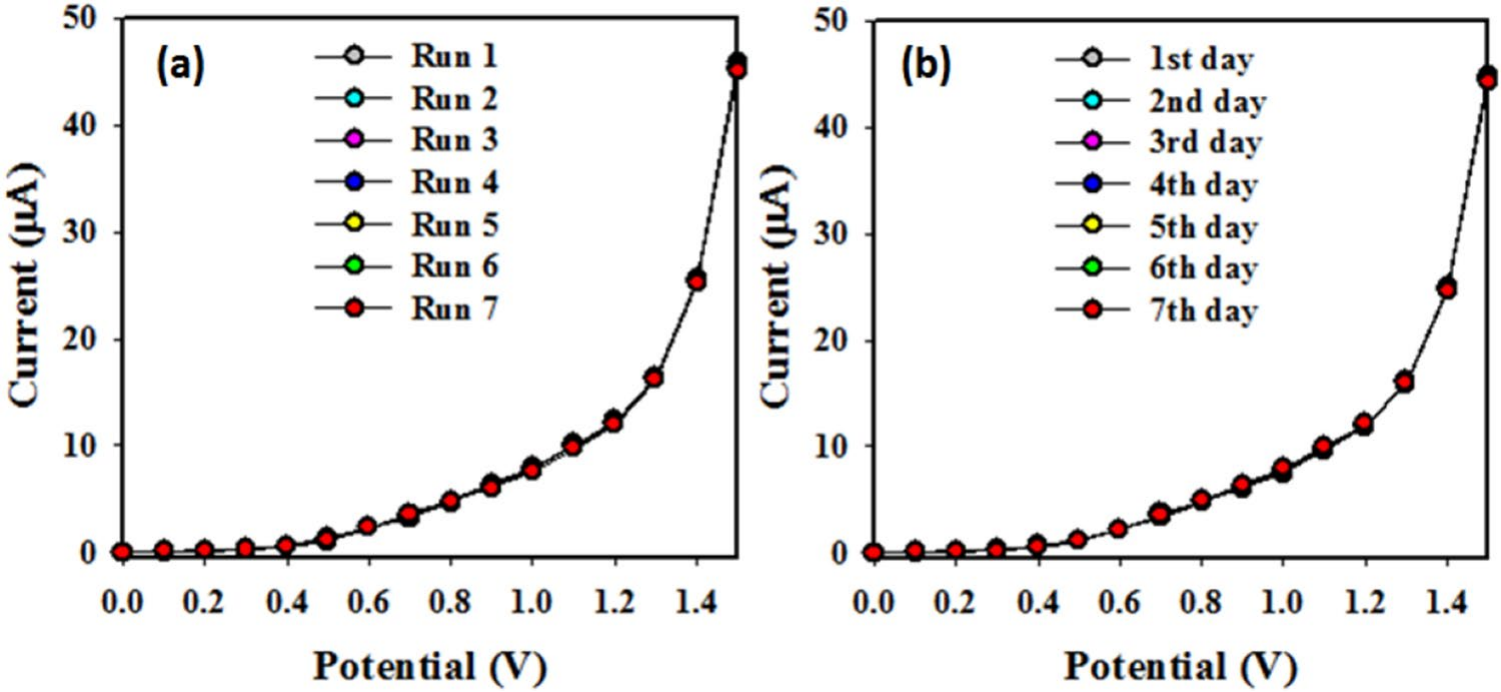
Optimization of CuO/MnO_2_/Gd_2_O_3_/binder/GCE sensor probe. (**a**) Reproducibility study and (**b**) Inter-day validity.

The materials fabricated CuO/MnO_2_/Gd_2_O_3_/binder/GCE sensor probe intra-day reproducibility (Fig. 8a) and inter-day validity (Fig. 8b) have been also studied and presented in Fig. 8. According to these studies, the sensor probe is reproduced almost the similar response in the same day in different measurements in the identical conditions. On the other hand, fabricated CuO/MnO_2_/Gd_2_O_3_/binder/GCE sensor probe is exhibited the almost similar reproducible responses in different inter-day measurement in the identical conditions, which is presented in the Fig. 8b.

**Figure 9:**
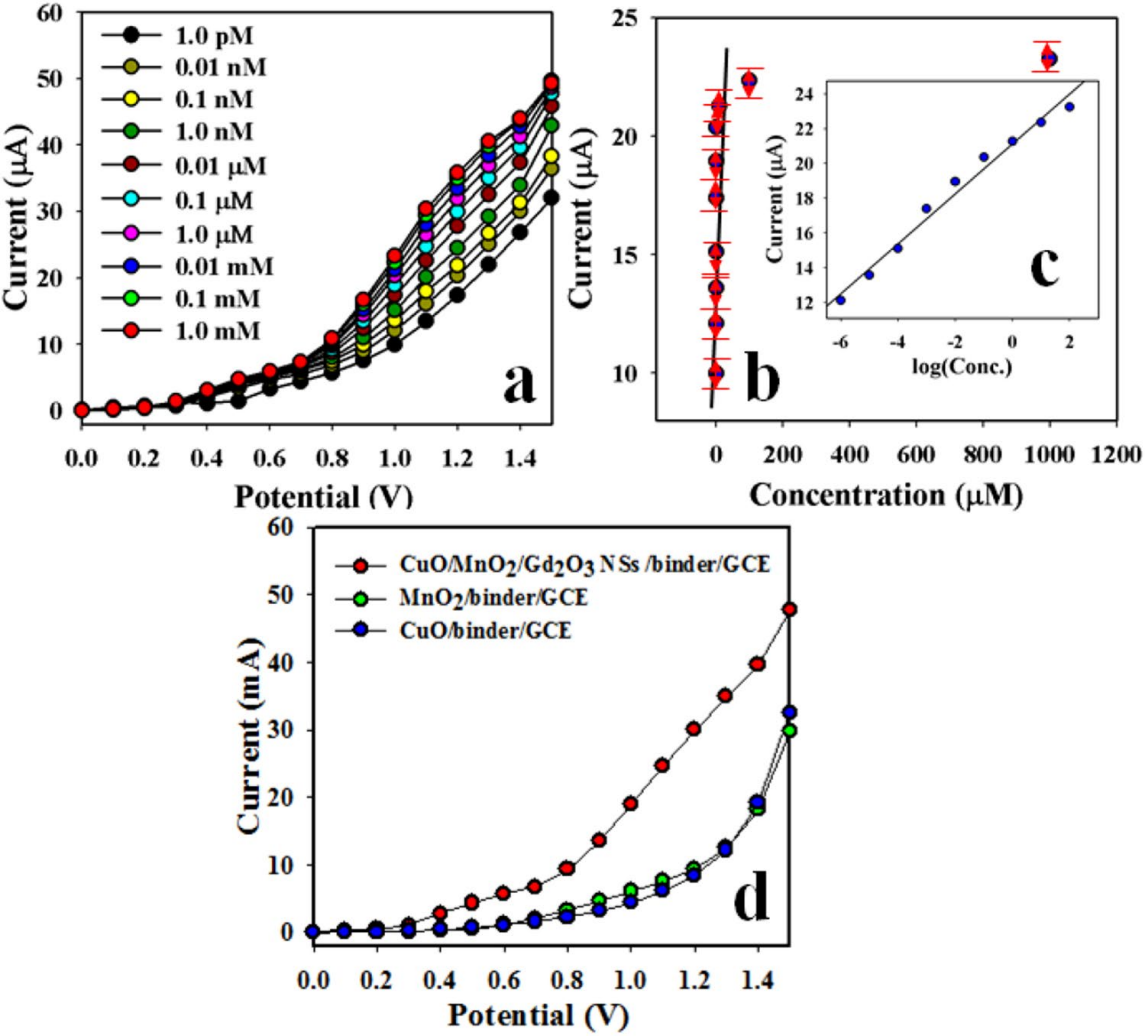
Detection of target chemical with the fabricated sensor probe. (**a**) Effect of concentration of 3-MPHyd compound based on CuO/MnO_2_/Gd_2_O_3_ NSs by electrochemical method, (**b**) calibration curve (Inset: log [3-MPHyd. Conc.] vs. Current), and (**d**) the comparison of electrochemical responses based on various electrodes. Analyte concentration: 0.1 nM; Holding time: 1.0 s; electrochemical method; Potential ranges: 0 to + 1.5.

As indicated in Fig. 9a, the electrochemical response of CuO/MnO_2_/Gd_2_O_3_ NSs/binder/GCE electrode is the maximum at the lowest concentration of 3-MPHyd. To execute this performance, a range of 3-MPHyd solution was prepared based on the concentration (full range 1.0 mM to 1.0 pM) and electrochemical measurement was carried out in the range from 0.0 to + 1.5 V. The linearity (r^2^: 0.9919) of the calibration plot (Fig. 9b,c was drawn as current versus concentration of 3-MPHyd). The analytical parameters have been calculated from the calibration plot (Fig. 9b) such as sensitivity (24.05 µA µM^−1^ cm^−2^), LOD (0.4 ± 0.02 pM at SNR of 3), LDR (1.0 pM to 0.1 mM), and Response time (9.25 s). Sensitivity was calculated from the slope of the calibration plot. It was calculated from the slope of the calibration plot by considering the active surface area of fabricated electrode (0.0316 cm^2^). During the sensing performances of 3-MPHyd chemical, the electrochemical current responses with CuO/MnO_2_/Gd_2_O_3_ NSs were increased with increasing of the target 3-MPHyd concentration. In presence of higher concentration of target analyte, the resultant current is gradually increased due to the oxidation of 3-MPHyd during electrochemical process with CuO/MnO_2_/Gd_2_O_3_ NSs. During the addition of analyte into the electrochemical solution, the oxidation current is increased gradually until 0.1 nM. After that the current response is found stable until 1.0 pM. No significant increase of current is occurred. A comparison between the electrodes fabricated on binary MnO/CuO, and ternary combinations of CuO/MnO_2_/Gd_2_O_3_ NSs were studied, and it was found that CuO/MnO_2_/Gd_2_O_3_ NSs/binder/GCE electrode was exhibited the highest electrochemical responses (Fig. 9d).

The possible reaction mechanism of the capturing under optimum condition is depicted in Fig. 5. The metal oxides nanomaterials are investigated as potential materials with various electroanalytical methods^57–59^. The significant application of CuO/MnO_2_/Gd_2_O_3_ NSs materials was employed in the capturing of environmentally toxic compound. The CuO/MnO_2_/Gd_2_O_3_ NSs materials are nontoxic, long-term stability, consistence, high electrochemical activity, nontoxicity and easy-to-use. The electrochemical method for the CuO/MnO_2_/Gd_2_O_3_ NSs is considerably changed during the adsorption of 3-MPHyd as the target agent. Here, Table 1, it is presented about the materials for capturing of hydrazine and their derivatives by various electrochemical approaches^60–72^. In this nano-formulated ternary CuO/MnO_2_/Gd_2_O_3_ NS materials have accomplished great deal of attention owing to their structural, chemical, optical, electrochemical, and morphological properties in terms of large-active surface area, high-stability as well as good porosity, and permeability^73–78^. This method has numerous benefits including easy and facile preparation, accurate control of the reactants temperature, easy to handle, one-step reaction, and high-porosity as well as porous natures^79–82^. Finally, this mixed CuO/MnO_2_/Gd_2_O_3_ NSs material have also attracted substantial attention owing to their impending applications in fabricating chemical devices, opto-electronics, electro-analytical, selective detection of chemical and biochemical assays, hybrid-composites, electron-field emission sources for emission exhibits, and biochemical detections etc.

**Table 1:** Performance of hydrazine and their derivatives compounds capturing performances with different nanomaterials by electrochemical method.

Capturing layer	Analyte	Methods	Performances	References
TiO_2_/CNT	Hyd	Amperometric	Sensitivity: 0.001497 µA cm^−2^ µM^−1^	^60^
			DL: 0.22 µM	
			LDR: 0.35–162 µM	
			Linearity, r^2^ = 0.993	
Ag/ZIF-8	Hyd	Amperometric	Sensitivity: 0.05446 µA cm^−2^ µM^−1^	^61^
			DL: 1.57 µM (at SNR of 3)	
			LDR: 6–5000 µM	
			Linearity, r^2^ = 0.998	
CNT powder microelectrode	Hyd	CV	Sensitivity: 0.9944 µA µM^−1^ cm^−2^	^62^
Ag-ZnO Nanoellipsoids	Hyd	CV	Sensitivity: 0.0946 mA µM^−1^ cm^−2^	^63^
			DL: 0.07 nM	
			LDR: 0.07–1.0 µM	
MWCNT and chlorogenic acid	Hyd	CV	Sensitivity: 0.0041 µA µM^−1^ cm^−2^	^64^
			DL: 8.0 nM	
Hierarchical micro/nanoarchitectures of ZnO	Hyd	CV	Sensitivity: 0.51 µA µM^−1^ cm^−2^	^65^
			DL: 0.25 nM	
			LDR: 0.8–200 µM	
Pristine ZnO NRs array	Hyd	CV	Sensitivity: 0.0448 mA µM^−1^ cm^−2^	^66^
			DL: 0.2 nM	
ZnO-II/Au	Hyd	CV	Sensitivity: 1.6 µA µM^−1^ cm^−2^	^67^
			DL: 0.066 nM	
			LDR: 0.066–425 µM	
ZnO/SWCNT	Hyd	CV	Sensitivity: 0.1 µAµM^−1^ cm^−2^	^68^
			DL: 0.17 nM	
			LDR: 0.5–50 µM	
ZnO Nanoflowers	Hyd	CV	Sensitivity: 0.0349 mA µM^−1^ cm^−2^	^69^
			DL: 0.18 nM	
Nano-Au ZnO-MWCNT	Hyd	CV	Sensitivity: 0.0428 µA µM^−1^ cm^−2^	^70^
			DL: 0.15 nM	
			LDR: 0.5–1800 µM	
ZnO/Yb_2_O_3_ nanosheets	Hyd	I–V	Sensitivity: 5.063 μA μM^−1^ cm^−2^	^71^
			DL: 0.019 ± 0.001 nM	
			LDR: 0.1 nM to 0.1 mM	
MnCo_x_O_y_ nanoparticles	Hyd	I–V	Sensitivity: 0.37 mA µmol L^−1^ cm^−2^	^72^
			DL: 0.26 ± 0.01 pmol L^−1^	
			LDR: 1.0 pmol L^−1^ to 1.0 µmol L^−1^	
CuO/MnO_2_/Gd_2_O_3_ NSs	3-MPHyd	I–V method	Sensitivity: 24.05 µA µM^−1^ cm^−2^	This work
			DL: 0.4 ± 0.02 pM (at SNR of 3)	
			LDR: 1.0 pM to 0.1 mM	
			Linearity, r^2^ = 0.9919	
			Response time: 9.25 s	

### Real sample analysis

The ternary CuO/MnO_2_/Gd_2_O_3_ NS materials fabricated electrode probe is potentiality depended on the real sample treating and others feasibility parameters^62–65^. To measure the 3-MPHyd in the real environmental sample with various concentrations, the fabricated material based on CuO/MnO_2_/Gd_2_O_3_ NSs was used to detect in the industrial effluent (collected from the Jeddah Industrial Area, Saudi Arabia) and extracted samples. The collected industrial effluent was initially filtered to remove the floating large particles and then filtered sample was directly used for analysis. The extracted sample from plastic baby bottle, plastic water bottle, and PVC food packaging bags were also filtered and analysed with CuO/MnO_2_/Gd_2_O_3_ NSs/Nafion/GCE sensor probe by electrochemical method. The analysis report is presented in the Table 2. The results clarified that the proposed NSs has high possibility to selective detection of 3-MPHyd significantly and efficiently.

**Table 2:** Analyses of 3-MPHyd into real environmental sample using CuO/MnO_2_/Gd_2_O_3_ NSs/Nafion/GCE sensor probe by electrochemical method.

Real sample	Added 3-MPHyd concentration (µM)	Determined 3-MPHyd concentration by CuO/MnO_2_/Gd_2_O_3_ NSs/GCE (µM)	Recovery (%)	RSD (%) (n = 3)
Industrial effluent	0.0100	0.01085	108.5	
	0.0100	0.01033	103.3	5.03
	0.0100	0.009811	98.11	
Plastic baby bottle	0.0100	0.009689	96.89	
	0.0100	0.009407	94.07	1.88
	0.0100	0.009743	97.43	
Plastic water bottle	0.0100	0.01145	114.5	
	0.0100	0.01190	119.0	2.55
	0.0100	0.01202	120.2	
PVC food packaging bag	0.0100	0.01028	102.8	
	0.0100	0.01035	103.5	0.37
	0.0100	0.01029	102.9	

## Conclusions

In this study, selective 3-MPHyd chemical material based on nano-formulated CuO/MnO_2_/Gd_2_O_3_ spike was fabricated and reported in details. The NSs of transition metal oxides were prepared by the hydrothermal approach in alkaline phase at low-temperature. The fabricated nanospikes were totally characterized by using FTIR, UV–Vis, XRD, XPS and FESEM. The slurry of ternary CuO/MnO_2_/Gd_2_O_3_ NSs was coated onto GCE as a layer of thin-film with conducting binder of nafion solution for selective and sensitive electrochemical detection of 3-MPHyd toxic compounds. The important experimental parameters such as sensitivity, low limit of detection, quantification, reaction time, sensitivity and reusability were performed systematically. The obtain results were good and satisfactorily enough to determine the target 3-MPHyd in short response time by electro-chemical approach. Here, CuO/MnO_2_/Gd_2_O_3_ NSs materials are an effective and potential for the selective detection of 3-MPHyd. Thus the materials can be used in broad scales for the efficient detection of selective 3-MPHyd by electrochemical method at room conditions for environmental remediation.
